# SSX2 regulates focal adhesion but does not drive the epithelial to mesenchymal transition in prostate cancer

**DOI:** 10.18632/oncotarget.9802

**Published:** 2016-06-02

**Authors:** Jordan E. Bloom, Douglas G. McNeel

**Affiliations:** ^1^ Department of Medicine, University of Wisconsin, Madison, WI, USA; ^2^ University of Wisconsin Carbone Cancer Center, University of Wisconsin, Madison, WI, USA

**Keywords:** SSX, prostate cancer, CTA, focal adhesion, EMT

## Abstract

Prostate cancer is the most commonly diagnosed malignancy for men in the United States. Metastatic prostate cancer, the lethal form of the disease, has a life expectancy of approximately five years. Identification of factors associated with this transition to metastatic disease is crucial for future therapies. One such factor is the SSX gene family, a family of cancer/testis antigens (CTA) transcription factors which have been shown to be aberrantly expressed in other cancers and associated with the epithelial to mesenchymal transition (EMT). We have previously shown that SSX expression in prostate cancers was restricted to metastatic tissue and not primary tumors. In this study, we have identified SSX2 as the predominant SSX family member expressed in prostate cancer, and found its expression in the peripheral blood of 19 of 54 (35%) prostate cancer patients, with expression restricted to circulating tumor cells, and in 7 of 15 (47%) metastatic cDNA samples. Further, we examined SSX2 function in prostate cancer through knockdown and overexpression in prostate cancer cell lines. While overexpression had little effect on morphology or gene transcript changes, knockdown of SSX2 resulted in an epithelial morphology, increased cell proliferation, increased expression of genes involved in focal adhesion, decreased anchorage independent growth, increased invasion, and increased tumorigenicity *in vivo*. We conclude from these findings that SSX2 expression in prostate cancer is not a driver of EMT, but is involved in processes associated with EMT including loss of focal adhesion that may be related to tumor cell dissemination.

## INTRODUCTION

Prostate cancer is the most commonly diagnosed malignancy of men in the United States [1]. The majority of newly diagnosed prostate cancer can be cured with radiation therapy or surgery [2, 3]. However, approximately 1/3 of patients following treatment with surgery or radiation therapy will recur and eventually progress to metastasis. Metastatic prostate cancer, and metastatic disease progressing beyond initial androgen deprivation therapy is lethal, with a life expectancy of approximately five years [4]. This is despite the approval of several new therapies within the last few years that have on average each extended survival by a few months [5–8]. The identification of proteins involved in prostate cancer metastasis that might serve as targets for new therapies is consequently of high relevance to this disease.

One potential target of interest is the SSX gene family. The SSX family of proteins are cancer-testis antigens (CTAs), a group of proteins whose normal expression is restricted to immune-privileged testis germline cells, but display aberrant heightened expression in many different types of cancer [9, 10]. These immune-privileged germ cell tissues lack HLA class I molecules [11]. Therefore in theory, any CD8^+^ T cell-targeted therapy directed towards a CTA should be specific for cancer cells, effectively ignoring germ cells or other normal somatic cells, making these particularly interesting targets for immune-based therapies [12]. We previously screened the sera of prostate cancer patients against an expression library of 29 CTA family members, and identified SSX2 as one of the most commonly recognized CT antigens [13]. Importantly, we have shown SSX proteins are expressed solely in metastases and not in primary prostate tumors [14].

Despite the interest of our group and others in the SSX family as therapeutic targets, the function of the SSX family of proteins is largely unknown. An understanding of its function and whether it has oncogenic activity given its expression in metastatic disease is therefore critical. SSX family members have been shown to possess two highly conserved domains: a Kruppel-associated box (KRAB) domain, and an SSX repression domain (SSXRD) [15]. Both regions have been shown to act as transcriptional repressors [16], however the targets of these domains remain unknown. SSX has two known binding partners, SSX2IP and RAB3IP [16], both of which are largely uncharacterized. However, SSX proteins have been found to be associated with the epithelial to mesenchymal transition (EMT) [17–19]. Cancer cells undergoing EMT have been shown to have greater invasive and proliferative capacity which can result in dissemination to distal organs, and to initiate metastases formation [20]. SSX knockdown in melanoma and osteosarcoma cell lines results in impaired cell migration, and down regulation of EMT associated genes [18]. Additionally, SSX was found to co-localize with EMT-associated proteins vimentin (*VIM*) and matrix metalloproteinase 2 (*MMP2*) [19]. SSX overexpression in a breast cancer cell line was shown to increase cell proliferation and repress the epithelial marker E-Cadherin (*CDH1*) [17]. Finally, SSX was found to be highly expressed in mesenchymal stem cells as compared to other CTAs [19]. However, there has been no evidence to date SSX is similarly involved with EMT in prostate cancer, and there has been no evaluation of its function in prostate cancer cells.

Our observation that SSX expression is confined to the metastases but not primary tumors suggested SSX may be similarly involved with EMT leading to metastasis formation in prostate cancer. This study aimed to further elucidate SSX's function and expression pattern in prostate cancer, and to specifically determine whether it is involved in prostate cancer EMT. We found through *in vitro* and *in vivo* studies that SSX2 is not a driver of EMT, however its loss leads to morphological changes and increases in proteins associated with focal adhesion.

## RESULTS

### SSX2 was the most frequently expressed SSX family member in prostate cancer metastases and in the peripheral blood of patients with recurrent prostate cancer

**Figure 1:**
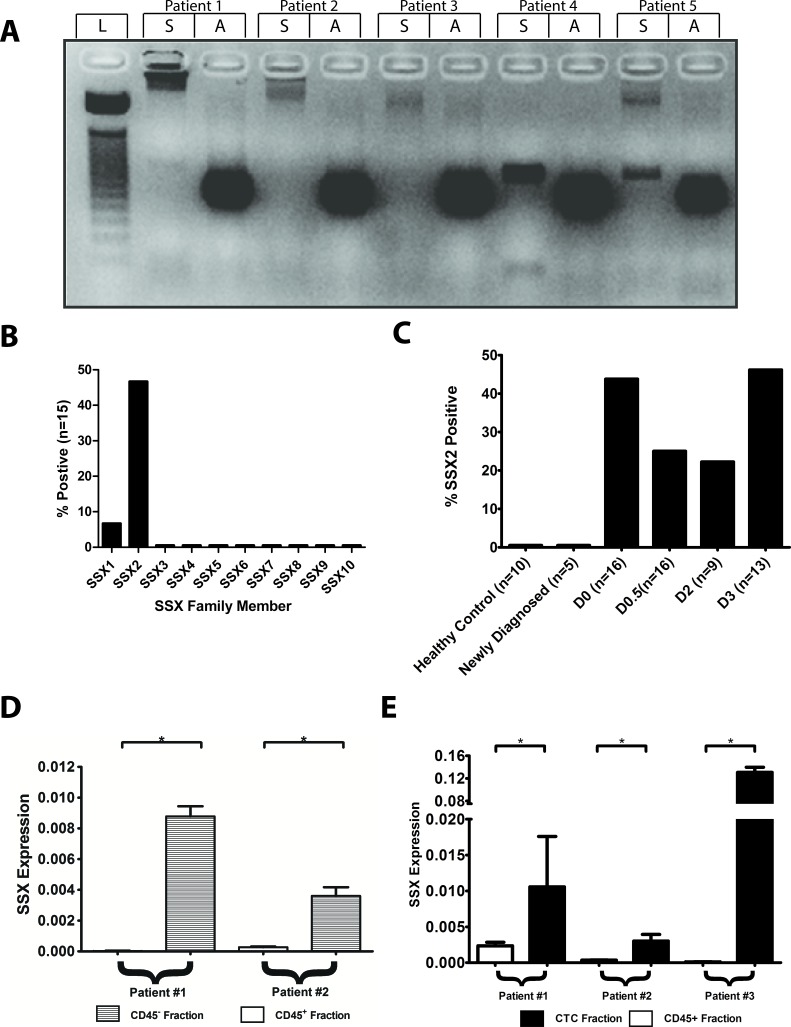
SSX2 is expressed in metastases and circulating tumor cells of prostate cancer patients cDNA libraries from 15 metastatic prostate cancer samples were evaluated for SSX gene expression using primers specific for each SSX family member. **A.** Representative agarose gel of SSX2 expression Key: S = SSX2, A = actin, L = DNA marker ladder. **B.** Summary of findings for all SSX family members in cDNA from metastatic tissues. **C.** SSX2 mRNA was detected in the blood of patients with recurrent prostate cancer by PCR using primers specific for SSX2. Key: D0 = non-castrate, non-metastatic; D0.5 = castrate-resistant, non-metastatic; D2 = castrate-sensitive, metastatic; D3 = castrate-resistant, metastatic. PBMC previously found positive for SSX2 expression were FACS sorted based on expression of cell surface markers. Quantification of SSX2 expression was performed in CD45^+^ or CD45^−^ populations **D.** and CD45^+^ or CD45^−^/EpCAM^+^/CD63^+^ (CTC) populations **E.** * = *P* < 0.05

The SSX family of proteins consists of 10 highly homologous members [21, 22]. Previous work has demonstrated through IHC of a tissue microarray that one or more SSX proteins were detectable in metastases but not primary prostate cancer tumors [14]. Given the homology among the SSX family members, the precise family member(s) expressed could not be determined in those studies. Therefore, we first evaluated metastatic tissues for the expression of each SSX family member by PCR. Using primers specific for each of the ten SSX family members [14], we screened cDNA obtained from 15 different prostate cancer metastases from different individuals (Figure 1A and 1B). SSX1 and SSX2 were detected in the metastatic samples at rates of 1 of 15 (6%) and 7 of 15 (47%) respectively (Figure 1B). Expression of the other SSX family members was not detected. The one sample with detectable SSX1 expression also had detectable SSX2 expression. Since SSX protein was not previously detected in primary tumors, and has been implicated in EMT, we next evaluated for the expression of SSX in cells in peripheral blood samples. SSX2 was the only family member detected in the peripheral blood, and overall detected in 19 of 54 (35%) patient blood samples (Figure 1C). Importantly, SSX2 expression was only found in patients with recurrent disease, however there was no association between prevalence of SSX2 expression and stage of recurrent disease, or serum PSA level (data not shown). Given these findings we concluded that SSX2 is the SSX family member most relevant to prostate cancer.

### SSX2 was detected in a CD45^−^/EpCAM^+^/CD63^+^ cell population in patient peripheral blood

Since we detected SSX2 mRNA in the peripheral blood of prostate cancer patients but not healthy controls, we assumed that the detection was of circulating tumor cells expressing SSX2, rather than, for example, cell-free tumor-associated RNA. Using fluorescence activated cell sorting (FACS), we separated cells into distinct populations of interest, then performed qPCR to analyze those populations for SSX2 expression. We found SSX2 expression was highly enriched in the CD45^−^ (non-hematopoietic) fraction, as compared to CD45^+^ control (Figure 1D). Furthermore, SSX2 was specifically enriched in the CD45^−^/EpCAM^+^/CD63^+^ subpopulation, which marks prostate-specific circulating tumor cells [23] while differentiating from erythroid progenitor CD45^−^/EpCAM^+^ cells (Figure 1E).

### Changes in SSX2 expression level were associated with non-canonical changes in EMT-associated genes

**Figure 2:**
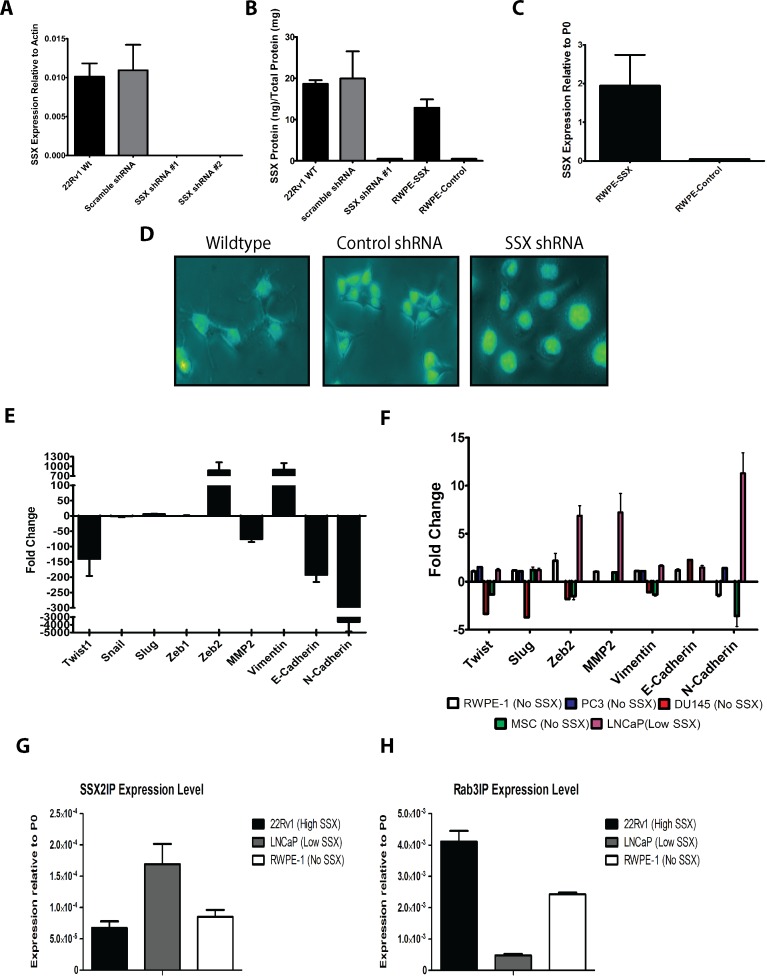
SSX2 modulated expression of EMT associated genes, but did not drive the EMT transition **A.** qPCR for SSX2 expression wild type (WT) and two different shRNA transfected and control (shRNA scramble) 22Rv1 cell lines. **B.** SSX2 protein quantification using ELISA in SSX shRNA and control cell lines. **C.** SSX2 expression by qPCR, relative to P0 housekeeping protein, in RWPE-1 cells transfected with empty vector or to express SSX2. **D.** 30x Microscopy images of Hoechst-stained shRNA transfected 22Rv1 cell lines or wildtype 22Rv1. **E.** Fold change by qPCR of EMT associated genes (Twist1, Snail, Slug, Zeb1, Zeb2, MMP2, Vimentin, E-Cadherin, N-Cadherin) in shRNA transfected 22Rv1 cell lines compared with scramble control transfected 22Rv1 cells. **F.** Fold change of EMT associated genes in cell lines transiently overexpressing SSX2 *vs* empty vector transfected control in different cell lines (MSC = human mesenchymal stem cell line). Expression levels of RAB3IP **G.** and SSX2IP **H.** genes in 22Rv1, LNCaP, and RWPE-1 cell lines by qPCR.

Previous studies in other malignancies have suggested a role for SSX in EMT [17–19]. Given the prevalence of SSX2 in the peripheral blood of patients with prostate cancer, we next questioned whether SSX2 expression in prostate cancer cells was similarly associated with markers of EMT. For these studies we took advantage of prostate cell lines that were previously characterized with respect to SSX2 expression [14]: 22Rv1 (a prostate cancer cell line with high SSX2 expression), LNCaP (a prostate cancer cell line with low SSX2 expression), DU145 and PC3 (prostate cancer cell lines with no SSX2 expression), and RWPE-1 (a prostate epithelial cell line with no SSX2 expression). Of note, 22Rv1 is an epithelial cell line and PC3 has a more mesenchymal phenotype, suggesting that SSX2 expression itself was not necessarily associated with a mesenchymal phenotype. To study changes in EMT associated with changes in SSX2 expression, we knocked down expression of SSX2 in the 22Rv1 cell line using an shRNA plasmid specific for SSX2, and verified by qPCR and ELISA (Figure 2A) and 2B). Conversely, the non-SSX2 expressing prostate cell lines (DU145, PC3, RWPE-1) were transfected with a mini-intronic plasmid encoding SSX2 to generate cells which ectopically overexpressed SSX2, and confirmed by qPCR and ELISA (Figure 2B and 2C). Upon transfection of the SSX knockdown plasmid, the 22Rv1 line began to exhibit a rounded morphology, less clumping, and “cobblestone” appearance (Figure 2D and Supplementary Figure 1). Upon overexpression of SSX2 in DU145, PC3, or RWPE lines, we observed no change in cell morphology (data not shown). We then evaluated genes with known EMT involvement by qPCR in the SSX2 knockdown and ectopically overexpressed cell lines (Figure 2E and 2F). SSX2 knockdown in the 22Rv1 line resulted in altered expression of EMT associated genes, but not in a canonical pattern typical of “drivers” of EMT (e.g. a “driver” would increase expression of Twist1 (*TWIST1)*, Zeb2 (*ZEB2*), Vimentin (*VIM*), Snail (*SNAI1*), Slug (*SNAI2*) and N-Cadherin (*CDH2)*, and decrease E-Cadherin (*CDH1)*). Rather, knockdown of SSX2 in the 22Rv1 line led to Twist1 being highly downregulated while Zeb2 and Vimentin were highly upregulated, and no change was seen in Snail or Slug (Figure 2E). Overexpression of SSX2 in the SSX negative prostate cell lines resulted in no or ambiguous changes in EMT associated genes. However, canonical changes in EMT-associated genes were detected following SSX2 overexpression in the LNCaP line (Figure 2F). Specifically, the transcription factor *ZEB2*, collagenase *MMP2*, and N-cadherin were upregulated in response to SSX2 overexpression (Figure 2F). These gene expression changes in the SSX2 overexpressing LNCaP line were not inversely related to the gene expression changes following knockdown of SSX2 expression in the 22Rv1 cell line, or seen in PC3 or DU145 cell lines. Taken together, these results suggested that SSX2, while associated with changes that occur with EMT, is by itself not a driver of EMT in the context of prostate cancer. The finding that SSX2 overexpression led to gene changes in one cell line but not another further suggested SSX2 may require additional cofactors for function. Therefore, we hypothesized one or both of SSX's known two binding partners, SSX2IP and Rab3IP, may have been responsible for EMT associated gene changes in the LNCaP but not RWPE-1. However, expression of these binding partners was detected within an order of magnitude in examined cell lines and did not appear to account for the differences observed following SSX2 knockdown or overexpression (Figure 2G and 2H).

### Microarray analysis revealed SSX2 has an association with focal adhesion

**Figure 3:**
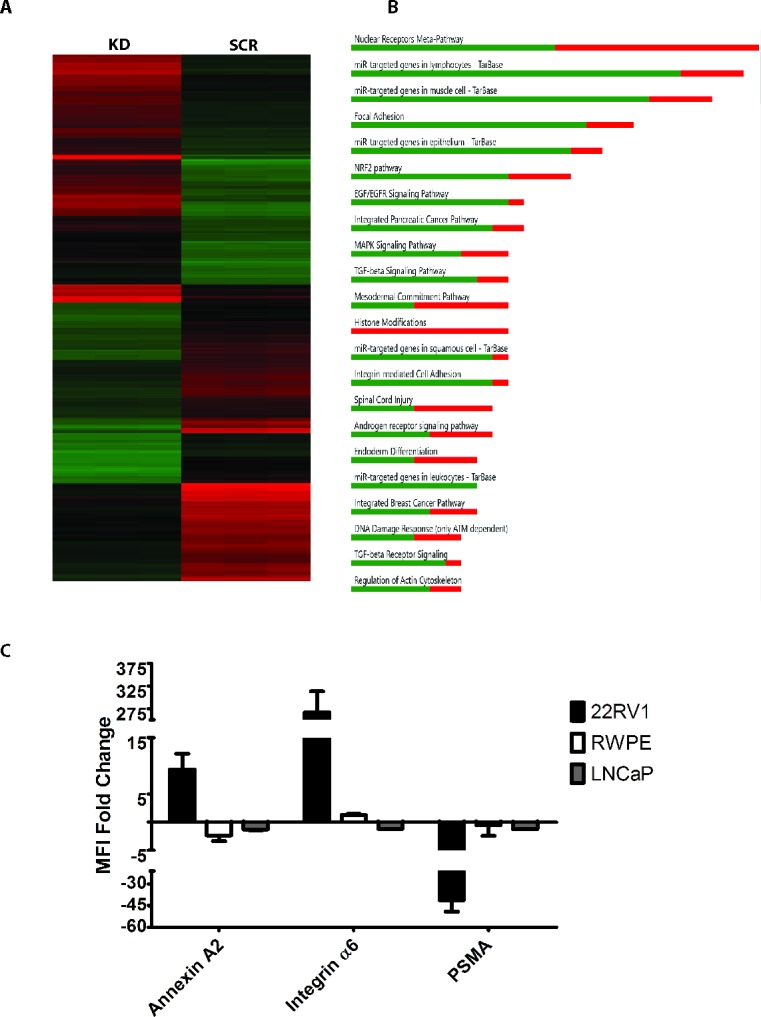
SSX knockdown showed changes in genes associated with focal adhesion **A.** Heat map of regulated genes in shRNA transfected (KD) and control (SCR) 22Rv1 cell lines (red = downregulated, green = upregulated), evaluated in triplicate samples each. **B.** Regulated genes by pathway in SSX shRNA *vs* scramble shRNA (red = downregulated, green = upregulated). **C.** Fold change of mean fluorescence intensity (MFI) of cell surface proteins found by microarray. For this analysis, change is evaluated with respect to mock transfection for 22Rv1 cells (knocked down for SSX2 expression) and RWPE-1 or LNCaP cells (overexpressing SSX2).

To assess other, non-EMT-related, functions of SSX2 in an unbiased fashion, we assessed global gene expression changes in the 22Rv1 SSX2 knockdown line compared to scramble shRNA or wildtype controls by gene microarray. Of 67,528 transcripts analyzed, 2,213 showed a significant decrease and 2,025 showed a significant increase following SSX2 knockdown compared to scramble shRNA control. When comparing the control cell lines (scramble control and wildtype) only 107 genes were found to be significantly changed, as compared to 4,238 between KD and SCR (Table 1 and Figure 3A). In particular, many genes related to focal adhesion were found to be upregulated including: *CADM1 ICAM1 COL12A1 EMP3 ITGA6* (Table 2 and Figure 3B). We confirmed these findings through flow cytometry by examining the expression of highly regulated cell surface proteins: Annexin A2 (*ANXA2)*, Integrin α6 (*ITGA6*), as well as PSMA (*FOLH1*), on SSX2 knockdown and scramble control 22Rv1 lines. Interestingly, the expression of these proteins was not reciprocally changed on RWPE-1 or LNCaP lines overexpressing SSX2 (Figure 3C).

**Table 1:** Genes with highest fold change following SSX shRNA 22Rv1 cell line from microarray

Fold Change (KD *vs*. SCR)	Gene Symbol	Description
81.65	ANXA2	annexin A2
66.37	ANXA1	annexin A1
65.07	MYOF	myoferlin
49.63	ANKRD1	ankyrin repeat domain 1 (cardiac muscle)
42	EDIL3	EGF-like repeats and discoidin I-like domains 3
33.01	ANXA3	annexin A3
32.26	UCHL1	ubiquitin thiolesterase
26.69	SLC36A4	solute carrier family 36 (proton/amino acid symporter), member 4
26.64	AXL	AXL receptor tyrosine kinase
25.6	RAB31	RAB31, member RAS oncogene family
−25.41	TPTE	transmembrane phosphatase with tensin homology
−25.43	SEMA6A	sema domain, transmembrane domain (TM), and cytoplasmic domain, (semaphorin) 6A
−26.17	FAR2	fatty acyl CoA reductase 2
−29.46	GPC6	glypican 6
−30.02	CKMT1A; CKMT1B	creatine kinase, mitochondrial 1A; creatine kinase, mitochondrial 1B
−31.48	SSX3	synovial sarcoma, X breakpoint 3
−36.78	HMGCS2	3-hydroxy-3-methylglutaryl-CoA synthase 2 (mitochondrial)
−37.59	SCG3	secretogranin III
−38.48	COLEC12	collectin sub-family member 12
−40.56	COLCA1	colorectal cancer associated 1
−42.29	POTEI	POTE ankyrin domain family, member I
−44.2	POTEI; POTEJ	POTE ankyrin domain family, member I; POTE ankyrin domain family, member J
−52.86	PTPRB	protein tyrosine phosphatase, receptor type, B
−54.29	POTEE	POTE ankyrin domain family, member E
−55.45	POTEF	POTE ankyrin domain family, member F
−63.35	AR	androgen receptor
−78.25	SLITRK6	SLIT and NTRK-like family, member 6
−78.75	FOLH1B; FOLH1	folate hydrolase 1B; folate hydrolase (prostate-specific membrane antigen) 1
−112.42	SSX2B; SSX2	synovial sarcoma, X breakpoint 2B; synovial sarcoma, X breakpoint 2
−150.18	EPHA3	EPH receptor A3

**Table 2:** Fold change of focal adhesion genes

Fold Change	Gene Symbol	Description
16.39	ITGA6	integrin, alpha 6
14.2	CAV1	caveolin 1, caveolae protein, 22kDa
11.13	THBS1	thrombospondin 1
9.74	MET	MET proto-oncogene
8.99	LAMB3; MIR4260	laminin, beta 3; microRNA 4260
7.75	AKT3	v-akt murine thymoma viral oncogene homolog 3
6.71	ITGA3	integrin, alpha 3
4.49	EGFR	epidermal growth factor receptor
4.43	FLNA	filamin A, alpha
4.15	SHC1	SHC transforming protein 1
3.99	LAMA3	laminin, alpha 3
2.28	VCL	vinculin
1.83	PAK1	p21 protein (Cdc42/Rac)-activated kinase 1
1.6	LAMA5; MIR4758	laminin, alpha 5; microRNA 4758
1.58	ZYX	zyxin

### Knockdown of SSX2 expression resulted in phenotypic and functional changes associated with cellular adhesion

**Figure 4:**
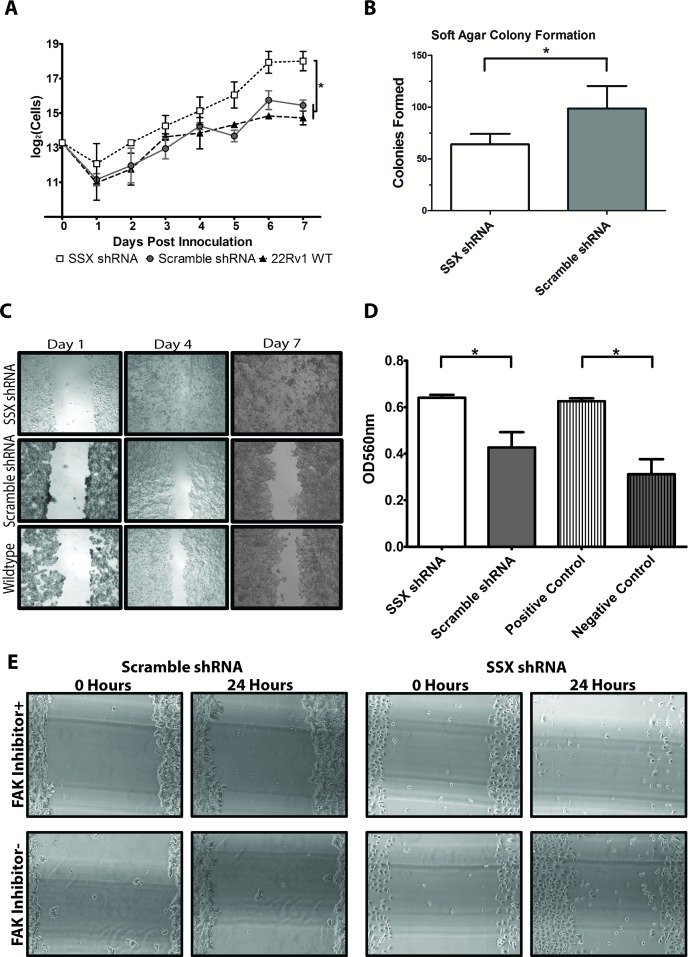
SSX2 knockdown resulted in functional changes in prostate cancer cells associated with adhesion and migration **A.** Quantification of cell proliferation in shRHA transfected and control 22Rv1 cell lines. **B.** Equivalent numbers of SSX shRNA and scramble shRNA 22Rv1 cell lines were plated in soft agar and colonies formed were counted after 7 days. **C.** 10x images of SSX shRNA and scramble shRNA 22Rv1 cell lines were grown to near confluence and a scratch introduced. Cells migrating into the space were imaged at 1, 4, and 7 days later. **D.** Equivalent numbers of SSX2 shRNA and scramble shRNA 22Rv1 cell lines were cultured in serum-free medium in the upper chamber of a transwell plate. Migration into the lower chamber was measured by OD_560_ after 48 hours. Positive and negative controls were as described in Methods. **E.** 10x images of extracellular filling “scratch” assays were conducted as in panel C in the presence or absence of FAK inhibitor 14. * = *P* < 0.05.

The 22Rv1 cell line, and derivatives transfected with SSX2 or scramble shRNA, was next evaluated for differences in phenotype and tumorigenicity. We first examined the growth rate of the different cell lines, and found SSX2 knockdown line demonstrated the highest growth rate while scramble control and wildtype lines were not significantly different (Figure 4A). We next investigated anchorage-independent growth of the SSX2 knockdown 22Rv1 line by colony formation in soft agar. The SSX2 knockdown line was less able to form colonies in the soft agar matrix as compared to scramble shRNA or wildtype control (Figure 4B). To phenotypically evaluate SSX2's effects on focal adhesion we investigated mechanisms reliant on focal adhesion [24–27]. Specifically, we found the SSX2 knockdown line both better able to fill new extracellular spaces (Figure 4C) and invade (Figure 4D) significantly better relative to shRNA scramble control. To specifically examine the effects of focal adhesion inhibition, we incubated SSX knockdown and scramble control with FAK inhibitor 14 and repeated the extracellular space filling “scratch” assay. FAK inhibition abrogated the phenotype seen in the SSX2 knockdown line (Figure 4C) and had no effect on shRNA scramble control cells (Figure 4E). To ensure the observed migration was not affected by changes in cell proliferation, migration and scratch test studies were also conducted in the presence of mitomycin C with similar findings (Supplementary Figure S2) [28]. Taken together, these data corroborate the finding that SSX2 knockdown increases focal adhesion and migration of a prostate cancer cell line.

### Knockdown of SSX2 expression leads to increased tumorigenicity *in vivo*

**Figure 5:**
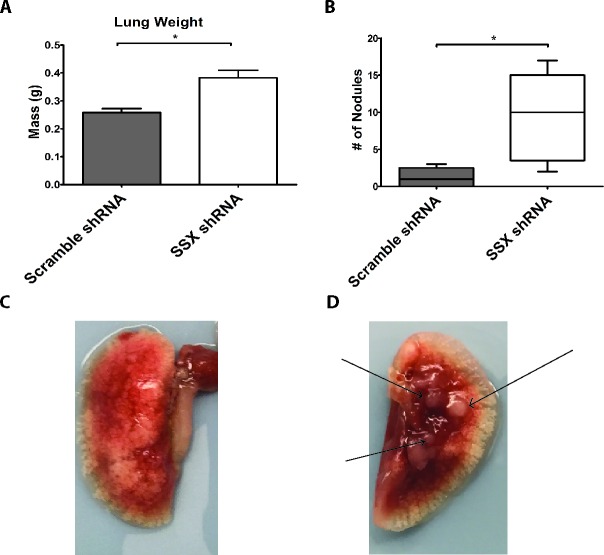
Knockdown of SSX increased tumor formation *in vivo* **A.** SCID mice were intravenously injected with equivalent numbers of SSX shRNA or scramble shRNA 22Rv1 cell lines and lungs harvested after 6 weeks. Shown are the lung weights **A.**, number of surface tumor lesions detected **B.**, and representative images **C.** with arrows pointing to tumor nodules. * = *P* < 0.05.

Due to SSX2's association with focal adhesion and cell migration, we next investigated the effects of SSX2 knockdown on tumor growth *in vivo*. SCID mice were intravenously injected with 1×10^6^ cells of either the SSX2 knockdown 22Rv1 cell line or scramble shRNA lines (Figure 5). At six weeks post injection the lungs were collected, weighed, and tumor nodules were counted. Mice injected with the SSX2 knockdown line were found to have significantly increased lung weights and more tumor nodules (Figure 5A and 5B).

## DISCUSSION

In this study we have shown SSX2 is the primary member of the SSX family expressed in prostate cancer, expressed in metastases and in circulating tumor cells. This expression pattern in metastases and in circulating tumor cells su­­ggests SSX2 could be functioning, or at least is expressed, in tumor cells with the capacity to disseminate. Using gene expression studies, we found SSX2 is involved with genes associated with EMT and specifically affects genes involved with cell adhesion. Upon knockdown of SSX2 in the 22Rv1 prostate cancer cell line we found that the SSX2 knockdown cells had less anchorage-independent growth, faster growth rate, increased invasion, and increased tumorigenicity in SCID mice. However, expression of SSX2 in prostate cancer cells was not associated with a mesenchymal phenotype, and forced overexpression in different prostate cell lines resulted in no phenotypic changes and no significant EMT-associated gene changes. These data indicate while SSX2 may be expressed in cancer cells undergoing EMT, and prostate cancer metastases, it is likely not an independent driver of EMT.

Given the high amino acid similarity of the SSX family of proteins, our previous study could not readily distinguish which SSX family members were expressed in metastatic prostate cancer using immunohistochemistry [14]. By using rtPCR we were able to demonstrate more definitively that SSX2 is the most frequently expressed member, while SSX1 is less frequently expressed (Figure 1A and 1B). Prior work from our lab has demonstrated that treatment of human prostate cancer cell lines with epigenetic modifying agents (EMAs) can lead to expression of other SSX family members, including SSX3, SSX5, and SSX8 [14]. However in this study, none of these were detected in untreated human tissue samples. In other diseases such as Hodgkin's lymphoma [29], multiple myeloma [30], and head and neck cancer [31] many different SSX family members are expressed. SSX1, SSX2, and SSX4 are expressed in Hodgkin's lymphoma and head and neck cancer, while SSX1, SSX2, SSX4, and SSX5 are expressed in multiple myeloma. In the case of multiple myeloma, expression of multiple SSX family members leads to worse prognosis and survival time [30]. These examples differ from prostate cancer where we predominantly saw only SSX2 expression, and there was no increase in the prevalence of SSX2 expression in PBMC from patients with more advanced stages of recurrent disease. Additionally, we found SSX2 is expressed in a CD45^−^/EpCAM^+^/CD63^+^ cell subset in the blood of patients with prostate cancer (Figure 1E), a cell subset specifically representing prostate-specific circulating tumor cells [23]. Thus, this is the first report of SSX2 expression in human CTC.

Our data indicate that SSX2 is involved in processes related to EMT in prostate cancer, notably focal adhesion, potentially accounting for the prevalence of expression in circulating tumor cells, but is itself not a driver of EMT. 22Rv1 cells that express SSX2 underwent a morphological change from a spiked appearance to a rounded epithelial appearance following SSX2 shRNA knockdown (Figure 2D) and S1). SSX2 knockdown, in the 22Rv1 prostate cancer line, demonstrated a large fold change in many different EMT associated genes (*TWIST1, ZEB2, MMP2, VIM, CDH1, CDH2*) however these genes did not respond in an anticipated or canonical way (Figure 2E). For example, both E-cadherin and N-Cadherin were down regulated, and the transcription factors Zeb2 and Twist1 were in opposition. Canonically, we expect EMT associated genes to move in the same direction when transitioning between epithelial to mesenchymal, and the cadherins to move in opposition (E-cadherin to decrease while N-cadherin increases, and vice versa). In fact, we found when knocking down SSX2 expression in the 22Rv1 line, N-cadherin expression was shut off while E-cadherin was down regulated. Curiously, while overexpression of SSX2 in the LNCaP cell line resulted in modest changes in EMT-associated genes (as expected), we saw low or no change in EMT genes following overexpression of SSX2 in SSX2 negative prostate cancer cell lines (Figure 2F). SSX's involvement with EMT was further supported by the microarray data of SSX knockdown in 22Rv1 cell line (Figure 3 and Supplementary Table S1). EPH receptor A3 (*EPHA3*) was found to have the highest negative fold change in the microarray. EPH receptors have been shown to be involved in the EMT pathway, and may regulate E-cadherin and Snail expression, as well as regulate MMP-2 activity [32–35]. Three different annexins were the highest upregulated genes when SSX was knocked down, and annexins have been shown to attenuate EMT in breast cancer [36] and regulate TGF-beta signaling [37].

Additionally, when we examined the effects of SSX2 knockdown on cellular proliferation (Figure 4A), we found increased cell proliferation. This observation is in opposition of those seen by D'Arcy et al [18], who found SSX knockdown inhibited proliferation in the melanoma DFW cell line, but in agreement with Chen, who found SSX-expressing breast cancer MCF-7 cells grew slower than a non-SSX expressing MCF-7 line [17]. Further, D'Arcy *et al* found E-cadherin downregulated in both DFW and SAOS-2 SSX-expressing melanoma and osteosarcoma cell lines, in agreement with our findings, but saw opposite changes in the expression of slug and vimentin [18]. We suspect these discrepancies are due to context-dependent factors related to different diseases or possibly co-factors expressed by different cell lines. These context-dependent factors are likely not the known binding partners of the SSX family (*RAB3IP* and *SSX2IP*) given our findings (Figure 2G and 2H). Hence, there is likely one or more undiscovered cofactor(s) that can function in tandem with SSX2. These data lead us to conclude SSX2 is associated with processes associated with EMT in prostate cancer, but SSX2 alone is not sufficient to drive EMT. SSX2 is known to associate with the polycomb-group complex proteins (PcG) [38] responsible for chromatin remodeling. Due to this association with PcG, methylation states between cell lines (or prostate cancer patients) may result in differential recruitment of needed cofactors, which could explain the observed context dependent differences. Further work should focus on identifying these context dependent factors.

Microarray analysis (Figure 3 and Table 1) revealed, in response to SSX2 knockdown, focal adhesion molecules such as *CADM1* (cell adhesion molecule 1), *ICAM1* (intercellular adhesion molecule1), *COL12A1* (collagen type 12), *EMP3* (epithelial membrane protein3), and *ITGA6* (integrin), were upregulated (Figure 3B and Table 1). The role of SSX2 in focal adhesion was further supported by studies demonstrating that loss of SSX2 led to increased invasion and extracellular space filling. In both assays the SSX knockdown line, with its heightened focal adhesion molecules, were able to invade or migrate more than the scramble shRNA or wildtype control lines (Figure 4C and 4D). Conversely, we find SSX2 knockdown resulted in decreased anchorage independent growth (Figure 4B). This suggests that when SSX2 is expressed in prostate cancer, focal adhesion molecules are downregulated. A decrease in focal adhesion could imply that factors leading to SSX2 expression aid in the extravasation from the primary tumor site into the blood stream and/or persistence of tumor cells in circulation.

Surprisingly, microarray analysis revealed knockdown of SSX2 also resulted in a knockdown of the androgen receptor (AR) and prostate specific membrane antigen (PSMA), both of which are intrinsically tied to prostate cancer disease progression [39, 40]. This was confirmed by evaluating surface expression of PSMA on these cells by flow cytometry (Figure 3C). Additionally, we examined the expression of the AR splice variants and similarly found them expressed at lower levels in response to SSX2 knockdown (Supplementary Figure S3). Castrate-resistant prostate cancer cells that acquire a completely AR-independent phenotype, whether of neuroendocrine or non-neuroendocrine type, typically display a more aggressive phenotype with rapid disease progression [41, 42]. Future research should investigate the relationship between SSX2 expression and AR-dependent growth.

Due to the high mortality of metastatic prostate cancer, therapeutic targets associated with advanced prostate cancer are needed. Because SSX2 is a CTA, and expressed in recurrent prostate cancer and not normal prostate cells, it is an attractive therapeutic target. The ability to specifically target metastatic tumor cells, and tumor cells in circulation without an immunosuppressive tumor microenvironment, could be advantageous. We have previously studied genetic vaccines targeting SSX2, and demonstrated that this is feasible [43]. Moreover, the absence of morphological or gene changes following overexpression in a normal epithelial cell line suggest this approach should not itself be oncogenic following expression of the gene in normal human cells. In fact, a therapeutic vaccine encoding SSX2 as one target antigen has recently opened to accrual for patients with advanced, metastatic prostate cancer (NCT02625857).

## MATERIALS AND METHODS

### Cell culture

Prostate cancer cell lines (LNCaP, 22Rv1, DU145, PC3) and an immortalized prostate epithelial cell line (RWPE-1) were grown in DMEM medium (CellGro/Mediatech, Manassas, VA) supplemented with 10% fetal calf serum (FCS)(Hyclone/GE, Logan, UT), 200U/ml penicillin/streptomycin (Thermofisher, Waltham, MA), 1 mM sodium pyruvate (Thermofisher, Waltham, MA), and 0.1μM β-mercaptoethanol at 37°C/5% CO_2_. All cell lines were validated by DDC Medical (Fairfield, OH), for identity and absence of mycoplasma contamination.

### Cell line plasmid DNA transfections

Cell lines of interest were plated in triplicate, and cultured to 80% confluence. Cells were then transfected with mini-intronic plasmid encoding SSX2, or empty vector control, using Lipofectamine^®^ LTX with Plus™ Reagent (Invitrogen, Carlsbad, CA). Cells were harvested after 3 days for RNA using Reliaprep RNA cell miniprep system (Promega, Fitchburg, WI). cDNA was generated using iScript™ cDNA Synthesis Kit (Bio-Rad, Hercules, CA) according to the manufacturer's instructions. SureSilencing™ shRNA plasmids against SSX2 were constructed by SAbiosciences (Frederick, MD). Transfected cells were then placed under neomycin selection, and SSX2 expression was verified using qPCR and ELISA as described below.

### Reverse transcription (RT)-PCR, and quantitative PCR analysis

RT-PCR using the One-Step RT-PCR kit (Qiagen, Valencia, CA) was carried out on RNA collected using Reliaprep RNA cell miniprep system (Promega) from peripheral blood samples from patients with prostate cancer or healthy control donors, under the following PCR conditions: 50°C for 30min.,95°C for 15min., 35 cycles at 95°C for 1 min., 60°C for 1 min. and 72°C for 1 min. Final extension for 10 min. at 72°C was followed by 4°C incubation until amplified products were resolved on a 2% agarose gel. Primers specific for each individual SSX family member and actin were previously identified [14]. cDNA generated from metastatic prostate tissue samples was provided by Dr. Robert Vessella (University of Washington, Seattle, WA). For quantitative PCR (qPCR), RNA was collected using Reliaprep RNA cell miniprep system (Promega) and reverse transcribed using iScript™ cDNA Synthesis Kit (Bio-Rad, Hercules, CA) according to the manufacturer's instructions. qPCR was performed using SsoFast™ EvaGreen^®^ Supermix (Bio-Rad) in a MyiQ™2 Two-Color Real-Time PCR Detection System (Bio-Rad) with annealing temperatures specific for each primer pair. All results were analyzed by the 2-^ΔCt^ method relative to P0 as a control gene [44]. The following analysis was used to determine fold change: 2-^ΔCt (Experimental)^/2-^ΔCt (Control)^. All primers used in this study are listed in Supplementary Table 1.

### Patient blood samples

With informed consent, peripheral blood or leukapheresis products were obtained from male subjects with (*n* = 59) or without prostate cancer (*n* = 10). Samples from patients with prostate cancer included men with newly diagnosed prostate cancer prior to prostatectomy (*n* = 5), recurrent prostate cancer with rising PSA after definitive therapy (stage D0/M0, *n* = 16), androgen-sensitive metastatic prostate cancer (stage D2, *n* = 9), non-metastatic castration-resistant prostate cancer (stage D0.5, *n* = 16), and metastatic castration-resistant prostate cancer (stage D3, *n* = 13). Peripheral blood mononuclear cells (PBMC) were isolated by Ficoll-Paque centrifugation (Pharmacia AB, Uppsala, Sweden) and cryopreserved in liquid nitrogen until use.

### ELISA

Detection of SSX2 in cell lysates was performed using a standard molecular biology sandwich ELISA with a capture monoclonal antibody specific for SSX2 (Abnova, Walnut, CA, Clone:1A4), and an SSX2 polyclonal detection antibody (Abnova, Walnut, CA).

### Soft agar colony formation

22Rv1 cells transfected with SSX2 shRNA or shRNA control, with SSX2 expression or knockdown confirmed via RT-PCR, were suspended in media with 0.343% agar (RPMI+10%FCS,2% Pen/Strep) and were then plated on a 0.6% agar bottom layer (RPMI+10%FCS,2% Pen/Strep, 1mg/ml G418). Initial cell inoculums were 1000 cells, and were plated in triplicate wells. After 2 weeks, wells were examined for colony formation, and colonies in a 1cm^2^ area were enumerated.

### Extracellular space-filling (“Scratch”) assay

Cells were plated in triplicate and grown in a 6 well dish. Scratches were made using a P200 pipette tip and imaged at 3 and 7 days after. Images are representative of the entire length of the scratch. To inhibit focal adhesion in this assay, 10μm of FAK Inhibitor 14 (Abcam Cambridge, MA) was added to the media at time of the scratch. To inhibit proliferation mitomycin C (Fisher) 10μg/ml was added to the media at the time of scratch.

### Cell proliferation

1×10^4^ 22Rv1 cells, or derivatives transfected with SSX2 shRNA or scramble shRNA control, with confirmed SSX2 expression, were plated in triplicate into a 24-well dish. Cell counts were made each day for 7 days using trypan blue staining to assess cell viability.

### Cell migration

A Boyden chamber assay (Cell Biolabs INC, San Diego, CA) was performed as per manufacturer's instructions using an FBS gradient. A positive control used cells seeded into the lower chamber, and the negative control was without the FBS gradient (no FBS in lower or upper chamber). To inhibit proliferation 10 μg/ml mitomycin C was added to the upper chamber.

### Microscopy

All Images were taken using a Nikon Eclipse Ti microscope and NIS-Elements D3.0 software. Magnification for each image is listed in the figure legend.

### Flow cytometry

Patient PBMC which tested positive for SSX2 mRNA by RT-PCR were washed with Hank's buffered saline solution and then stained with antibodies specific for CD45-FITC(clone:30-F11 BD, Franklin Lakes, NJ), EpCAM-PE (clone: EBA-1 BD), CD63-APC (clone:MEM-259 Biolegend, San Diego, CA) and DAPI (Molecular probes/ThermoFisher, Waltham, MA). Stained cells were analyzed and sorted using a FACS Aria (BD, Franklin Lakes, NJ). Circulating tumor cells (gated as: DAPI^−^/Singlet^+^/CD45^−^/EpCAM^+^/CD63^+^) or control lymphocytes (DAPI^−^/Singlet^+^/CD45^+^) were sorted using a BD FACS Aria into microfuge tubes containing BL lysis buffer (Promega). RNA and subsequently cDNA was generated and then assayed for SSX2 using qPCR as described above. Flow cytometry data was analyzed using FlowJo software version 10.

To measure levels of relevant surfaces markers found by the gene array, cells of interest were stained with Annexin A2-PE(clone: D11g2, Cell Signaling Technologies, Danvers, MA), Integrin alpha6-FITC(Clone:GoH3, Biolegend), and PSMA-PE-Cy7(clone:LNF-17, Biolegend). MFI was determined by comparison with IgG control using the FlowJo software. Fold change was then calculated by: MFI_experimental_/MFI_control_ and done in triplicate.

### Gene array

cDNA was generated and hybridized to an Affymetrix Human Transcriptome Array 2.0 (Affymetrix Santa Cruz, CA). Samples were normalized by RMA algorithm to a log2 intensity value and were analyzed by Transcriptome Analysis Console v3.0. Genes showed at least fivefold increased or decreased expression are shown after analysis using the Bonferonni correction. A one-way ANOVA test was used to determine significance between SSX knockdown expression profile to the scramble or wildtype control. These data have been deposited in NCBI's Gene Expression Omnibus [45] and are accessible through GEO Series accession number GSE77811 (https://www.ncbi.nlm.nih.gov/geo/query/acc.cgi?acc = GSE 77811).

### *In vivo* metastases formation in SCID mice

The protocol used was adapted from Mohanty and Xu [46]. Briefly, 10^6^ SSX knockdown or scramble control 22Rv1 cells were injected intravenously into CB17-Prkscid mice (Jackson Laboratories, Bar Harbor, ME). Mice (*n* = 5 per group) were euthanized after 6 weeks, with collection of lungs for weights and histological analysis. The harvested lungs were collected into 10% formalin and metastatic nodules on the surfaces of each lung were counted.

### Statistical analysis

The Student's T Test was used for comparisons, unless otherwise noted, with *p* < 0.05 considered as statistically significant.

## SUPPLEMENTARY MATERIALS FIGURES AND TABLES


