# Supplementary material for: SSX2 regulates focal adhesion but does not drive the epithelial to mesenchymal transition in prostate cancer
Source: Oncotarget. 2016 Jun 2;7(32):50997–1011. doi: 10.18632/oncotarget.9802 (PMC5239454; doi:10.18632/oncotarget.9802)
Supplement: Supplementary file 1 [file oncotarget-07-50997-s001.pdf]

SSX2 regulates focal adhesion but does not drive the epithelial to mesenchymal transition in prostate cancer

Supplementary Material

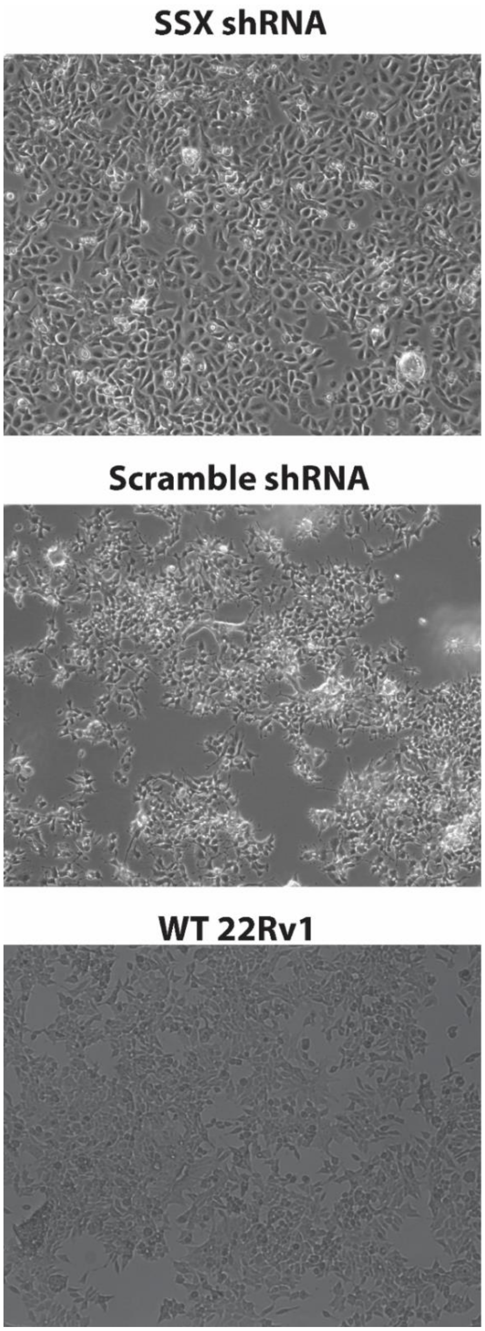

Supplementary Figure 1: <u>10x View of 22Rv1 cell lines</u>

10x view of 22Rv1 lines investigated in this study to demonstrate morphology.

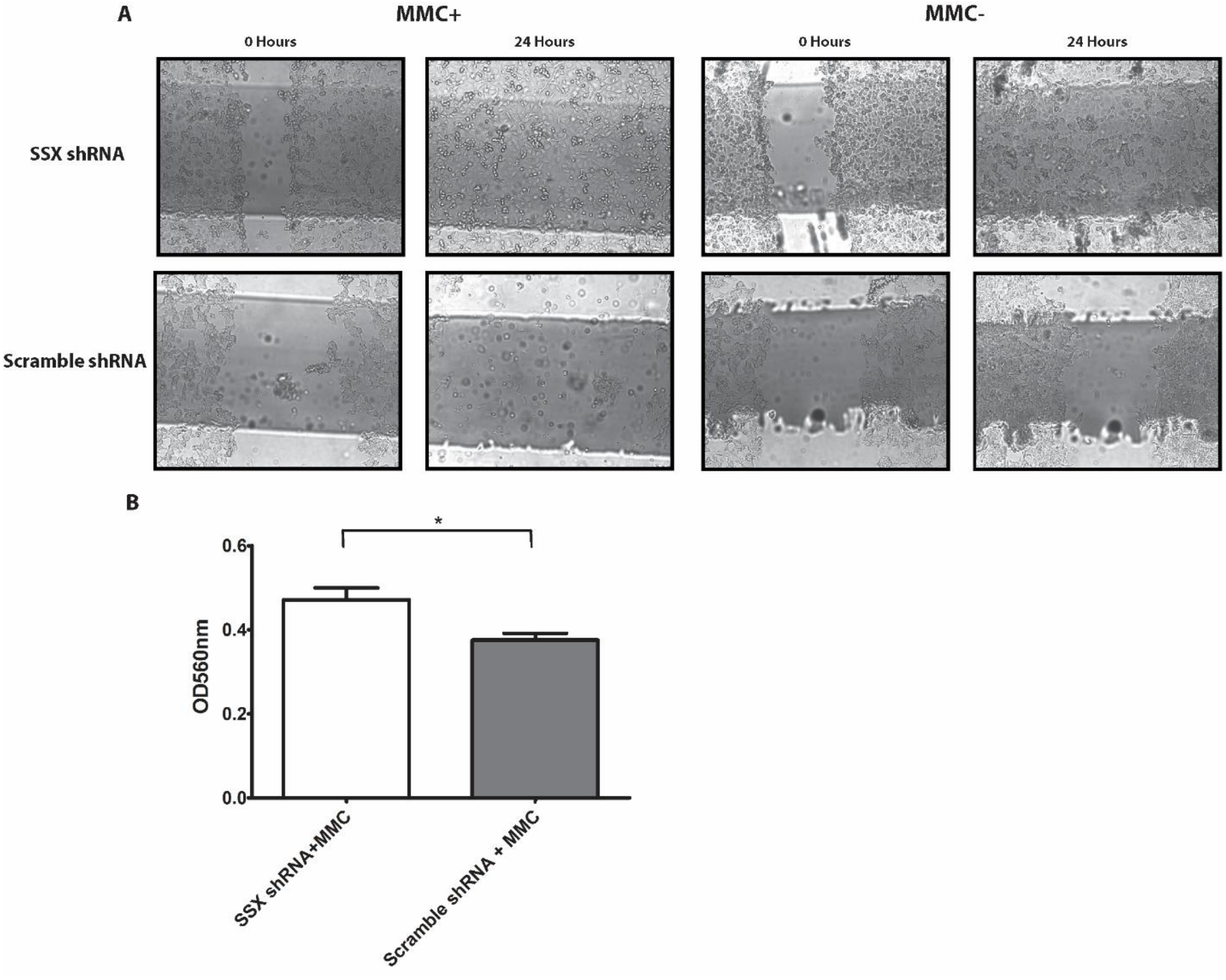

Supplementary Figure 2: <u>Migration in SSX knockdown in 22Rv1 is not affected by proliferation</u>

Migration studies were repeated in the presence of Mitomycin C (MMC). A. Scratch migration assay on SSX shRNA and scramble shRNA 22Rv1 lines in the presence or absence of MMC after 24 hours. B. Boyden chamber migration on SSX shRNA and scramble shRNA 22Rv1 lines in the presence or absence of MMC

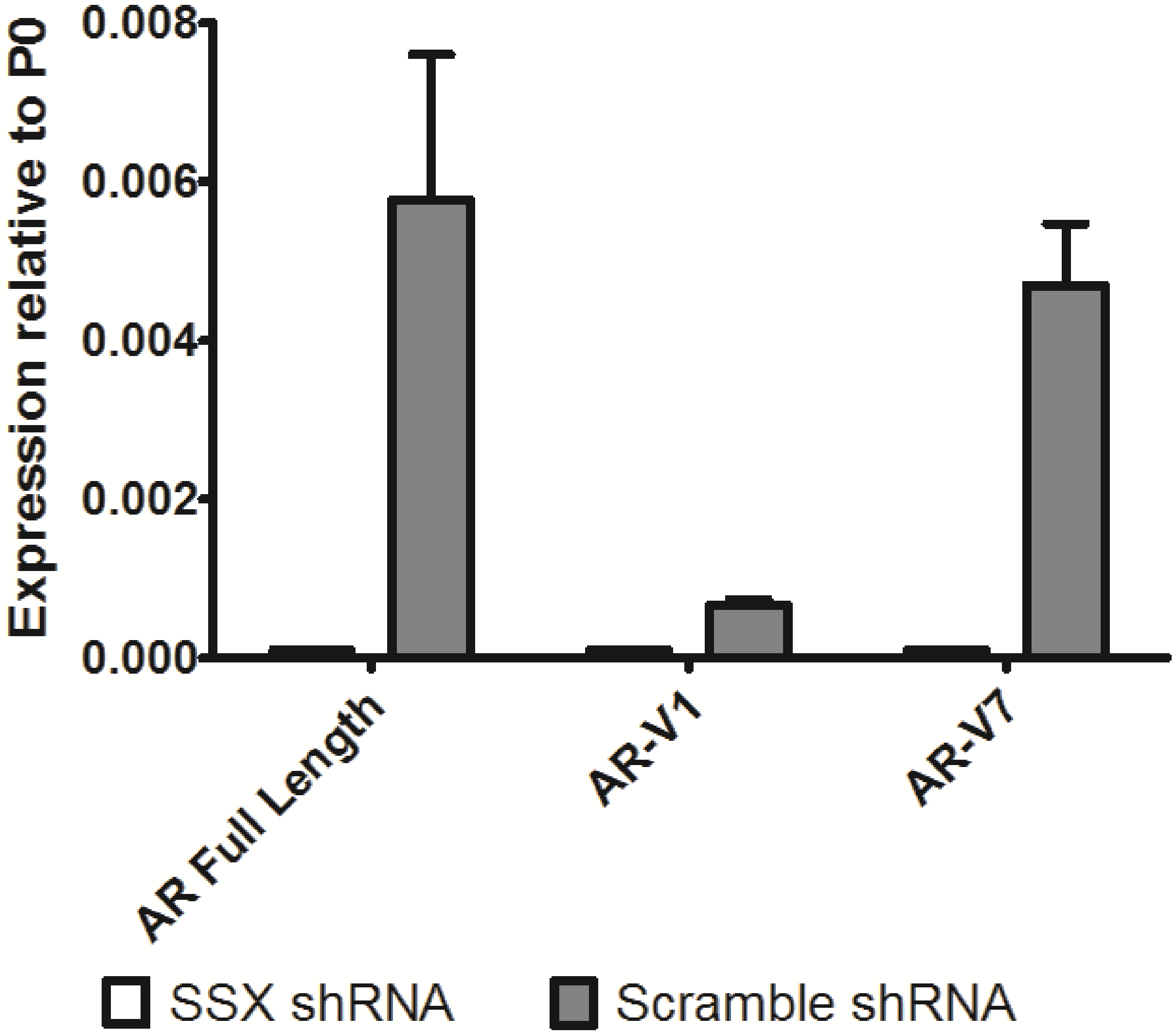

Supplementary Figure 3: Expression of AR and splicing variants in SSX shRNA and sxramble shRNA 22Rv1 lines

Expression levels of the androgen receptor (AR) and two of its known splice variants AR-V1 and AR-V7, in SSX2 shRNA and Scramble shRNA 22Rv1 cell lines.

Supplementary Table 1: Table of primers utilized in this study

Gene Target	Gene Symbol	Forward (5' to 3')	Reverse (5' to 3')
Twist1	TWIST1	GGAGTCCGCAGTCTTACGAG	TCTGGAGGACCTGGTAGAGG
Snail	SNAI1	GCTGCAGGACTCTAATCCAGA	ATCTCCGGAGGTGGGATG
Slug	SNAI2	TGGTTGCTTCAAGGACACAT	GTTGCAGTGAGGGCAAGAA
Zeb1	ZEB1	GATGATGAATGCGAGTCAGATGC	ACAGCAGTGTCTTGTTGTTGTAG
Zeb2	ZEB2	AACAACGAGATTCTACAAGCCTC	TCGCGTTCCTCCAGTTTTCTT
MMP2	MMP2	ATAACCTGGATGCCGTCGT	AGGCACCCTTGAAGAAGTAGC
Vimentin	VIM	AAAGTGTGGCTGCCAAGAAC	AGCCTCAGAGAGGTCAGCAA
E-Cadherin	CDH1	AGTGTCCCCCGGTATCTTCC	CAGCCGCTTTCAGATTTTCAT
N-Cadherin	CDH2	ACAGTGGCCACCTACAAAGG	CCGAGATGGGGTTGATAATG
P0	36B4	GACAATGGCAGCATCTACAAC	GCAGACAGACACTGGCAAC
SSX2qPCR	SSX2	ACGGTTGGTGCTCAAATACCAGAGAA	GGCATGATCTTCGGGGAGATTCC
AR full length	AR	ACATCAAGGAACTCGATCGTATCATTGC	AGCTTCTGGGTTGTCTCCTCAGTGG
AR-V1	AR	CCATCTTGTCGTCTTCGGAAATGTTATGAAGC	CTGTTGTGGATGAGCAGCTGAGAGTCT
AR-V5	AR	CCAAGGCCTTGCCTGATTGC	TTGGGCACTTGCACAGAGAT
